# Real-Time ATP Imaging Reveals the Metabolic State during Branching Nephrogenesis

**DOI:** 10.34067/KID.0000001148

**Published:** 2026-01-26

**Authors:** Akiko Mii, Shinya Yamamoto, Masamichi Yamamoto, Shigenori Yamamoto, Shingo Fukuma, Akira Shimizu, Hiromi Imamura, Motoko Yanagita

**Affiliations:** 1 Department of Nephrology, Graduate School of Medicine, Kyoto University, Kyoto, Japan; 2 Department of Human Health Sciences, Graduate School of Medicine, Kyoto University, Kyoto, Japan; 3 Department of Analytic Human Pathology, Nippon Medical School, Tokyo, Japan; 4 Graduate School of Biostudies, Kyoto University, Kyoto, Japan; 5 Institute for the Advanced Study of Human Biology (WPI-ASHBi), Kyoto University, Kyoto, Japan

**Keywords:** kidney development, malnutrition, nephron development

## Abstract

**Key Points:**

- Real-time *ex vivo* ATP imaging revealed spaciotemporal ATP dynamics of metanephric kidneys during nephrogenesis.
- Glycolytic inhibition reduced cytosolic ATP levels in ureteric bud and cap mesenchyme cells and suppressed ureteric bud branching.
- Ureteric bud cells were highly dependent on glycolysis, exhibiting faster and more pronounced ATP reduction by glycolytic inhibition than cap mesenchyme cells.

**Background:**

Energy metabolism is fundamental to organ development, yet its role in determining nephron formation remains poorly understood. Disturbances in the intrauterine environment are known to affect nephron endowment and increased risk of hypertension and CKD later in life. However, very little is known about the mechanisms that determine nephron number and metabolic status during nephrogenesis.

**Methods:**

We focused on cytosolic adenosine 5-triphosphate (ATP) levels to examine energy metabolism in embryonic kidneys. To explore the spatiotemporal dynamics of the cytosolic ATP level, we used transgenic mice expressing a cytosolic ATP-fluorescence energy transfer biosensor, GO-ATeam2, which enabled *ex vivo* live imaging of metanephric kidneys at single-cell resolution.

**Results:**

We performed real-time *ex vivo* ATP imaging of embryonic kidneys of GO-ATeam2 mice. During branching nephrogenesis, ATP levels of ureteric bud (UB) tip cells are significantly lower than those of the UB stalk and cap mesenchyme (CM) cells. Glycolytic inhibition in the early phase of metanephric kidney (embryonic day E12.5) severely suppressed UB branching with a dose-dependent reduction in ATP levels in both UB and CM cells. Time-course observations revealed that the ATP reduction by glycolytic inhibitor was faster and more prominent in UB cells than in CM cells. In addition, glycolytic inhibition significantly reduced the number of branch segments and tips of UB as well as the expression of specific markers in UB and CM cells. Electron microscopy revealed loosening of lateral cell-cell adhesion and disorganized alignment of CM cells, which were accompanied by decreased expression of N-cadherin. These effects were not observed with the inhibition of oxidative phosphorylation.

**Conclusions:**

UB branching was heavily dependent on glycolysis, and UB cells in the early branching phase were more sensitive to glycolytic inhibition than mesenchyme cells are. These results highlight the significance of metabolic regulation in branching nephrogenesis.

## Visual Abstract

**Figure:**
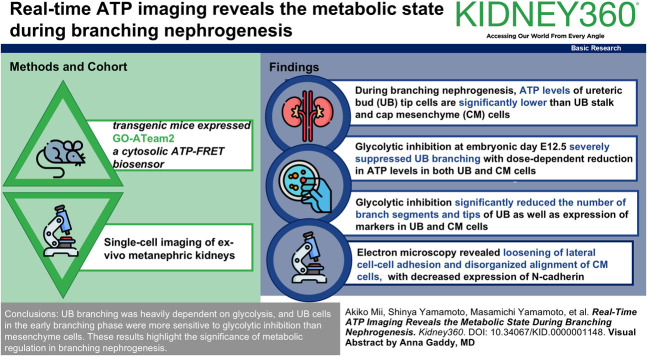

## Introduction

Many epidemiological studies have suggested that the intrauterine environment is important in determining an individual's future health.^1–4^ In humans, most of the nephrons are formed in the third trimester of pregnancy,^5,6^ and new nephrons are not formed after birth. Even in rodents, nephron formation ceases within several days of birth.^7^ Thus, the maternal environment is assumed to have a profound effect on the development of the mammalian kidneys. The developmental origins of health and disease is a concept that has emerged over the past 40 years, linking the environmental conditions during embryonic or early childhood to health status and disease risk later in life.^8–11^ Maternal malnutrition encompasses all forms of nutritional imbalance, including undernutrition, deficiencies of essential micronutrients, and overnutrition, such as overweight and obesity, and is considered one of the most significant risk factors for impaired fetal growth and a higher prevalence of low birth weight.^12,13^ Previous studies have shown that low birth weight and small size for gestational age are associated with reduced nephron numbers and an increased risk of hypertension and CKD in later life.^14–16^ Data obtained from animal studies also support this hypothesis, as is the case with human data.^17–21^ These data suggest that maternal malnutrition interferes with kidney development, leads to a low nephron number, and likely progresses to CKD. However, the precise mechanisms linking environmental or metabolic disturbances during development to reduced nephron number remain unclear.

The intricate differentiation process of the mammalian kidney arises from the reciprocal interactions of two distinct tissues of intermediate mesodermal origin, the ureteric bud (UB) and metanephric mesenchyme.^22^ The former differentiates into the collecting duct, whereas the latter forms nephron epithelium. Around embryonic day (E)10.5–11.0, the UB cells outgrow from the Wolffian duct toward the mesenchyme, and the mesenchyme around the UB aggregates to form cap mesenchyme (CM). CM subsequently induces UB branching, generating a tree-like network of the collecting duct.^23,24^ Concurrently, the branching tip of the UB induces epithelialization of mesenchymal cells, leading to the formation of the nephron epithelium. Recent reports have suggested that the glycolytic pathway in nephron progenitor cells (NPCs) plays an important role in maintaining their self-renewal capacity.^25,26^ However, there is no detailed analysis of the metabolic profiles during kidney development, especially in branching morphogenesis of UB, which also defines nephron number.^27^ Therefore, elucidating the spatial and temporal regulation of cellular energy metabolism during nephrogenesis is essential for understanding how metabolic programs influence kidney development.

ATP is a ubiquitous energy currency in all living organisms. The high phosphate transfer potential of ATP is used in many biological processes including cell proliferation, differentiation, membrane transport, and protein synthesis.^28^ During organ development, cells adopt distinct metabolic strategies to support growth, produce energy, and meet the demands of the mature tissues. Therefore, understanding intracellular ATP dynamics in organogenesis is crucial; however, the lack of technology to visualize ATP dynamics noninvasively has hindered further understanding. Conventional ATP quantification methods using luciferase assays can only provide the average ATP level of a cell population on the basis of the analysis of cell extracts. Mass imaging techniques provide the spatial distribution of ATP, but not ATP dynamics at a single-cell resolution. In addition, the recently invented system of Extracellular Flux Analyzer, which allows the analysis of metabolic profiles of cultured cells,^29^ is unable to assess spatiotemporal cytosolic ATP dynamics of distinct cell populations *in vivo*.

Imamura *et al*. created a series of fluorescence energy transfer (FRET)–based indicators for ATP called GO-ATeam.^30^ Subsequently, we generated a novel mouse line systemically expressing GO-ATeam by inserting cytomegalovirus (CMV) immediate-early enhancer and chicken β-actin promoter promoter-driven GO-ATeam in the ROSA26 locus (GO-ATeam2 mice).^31–34^ The mice allowed us to visualize and evaluate cytosolic ATP levels quantitatively using a two-photon microscopes. On the basis of this technical advance, we hypothesized that distinct cell populations exhibit differential intracellular ATP dynamics that reflect their metabolic state and contribute to the regulation of branching nephrogenesis. To test this hypothesis, we aimed to define the spatial and temporal characteristics of ATP dynamics during kidney development using real-time imaging.

In this study, using embryonic kidneys of GO-ATeam2 mice, we visualized and analyzed cytosolic ATP levels using real-time *ex vivo* imaging during branching nephrogenesis for the first time. We also revealed the effect of glycolysis and oxidative phosphorylation (OXPHOS) inhibition on ATP production during nephrogenesis.

## Methods

### Animals

GO-ATeam2 mice were generated in our laboratory and are described in a separate paper.^31^ Mice were housed in a specific pathogen-free facility and fed a standard diet.

Female C57BL/6J pregnant mice (wild-type mice) were used for the experiments without ATP imaging.

### Kidney Explant Culture and Reagents

Kidney explant cultures were performed as previously described.^35^ Kidney explants were isolated from pregnant wild-type/GO-ATeam2 mice and cultured on Millicell-CM Biopore membranes (pore size 0.4 *µ*m; Millipore, Billerica, MA) floating on DMEM supplemented with 10% FBS at 37°C in 5% CO_2_. 2-Deoxy-D-glucose (2-DG), a glycolysis inhibitor, was dissolved in DMEM and diluted in the culture medium. Rotenone, a mitochondrial complex 1 inhibitor that blocks OXPHOS, was dissolved in DMSO and diluted in culture medium.

### ATP Imaging by Two-Photon Microscopy

Isolated metanephric kidneys were cultured on Millicell-CM Biopore membranes in a 35 mm glass-based dish (AGC Techno Glass Co., Ltd., Shizuoka, Japan) floating culture solution. The dishes were placed inside a culture chamber in a humidified atmosphere containing 5% CO_2_ at 37°C. Time-lapse imaging was performed using an inverted two-photon microscope (LCV110-MPE, Olympus). In brief, we used an infrared-cut filter (BA750RXD), two dichroic mirrors (DM505 and DM690), and two emission filters (BA495–540 [Olympus] for green fluorescent protein [GFP] and BA562–596 [Olympus] for Kusabira orange fluorescent protein [KO], respectively). Cells were excited at a wavelength of 930 nm. The fluorescence images of GFP and KO, the latter of which was excited by FRET, were simultaneously acquired. In the GO-ATeam2–expressing cells, the FRET/GFP fluorescence intensity ratio in each pixel correlates with the cytosolic ATP level,^32–34^ thereby allowing ATP monitoring.

### Analysis of Ratio Changes in CM and UB Cells

**Figure 1:**
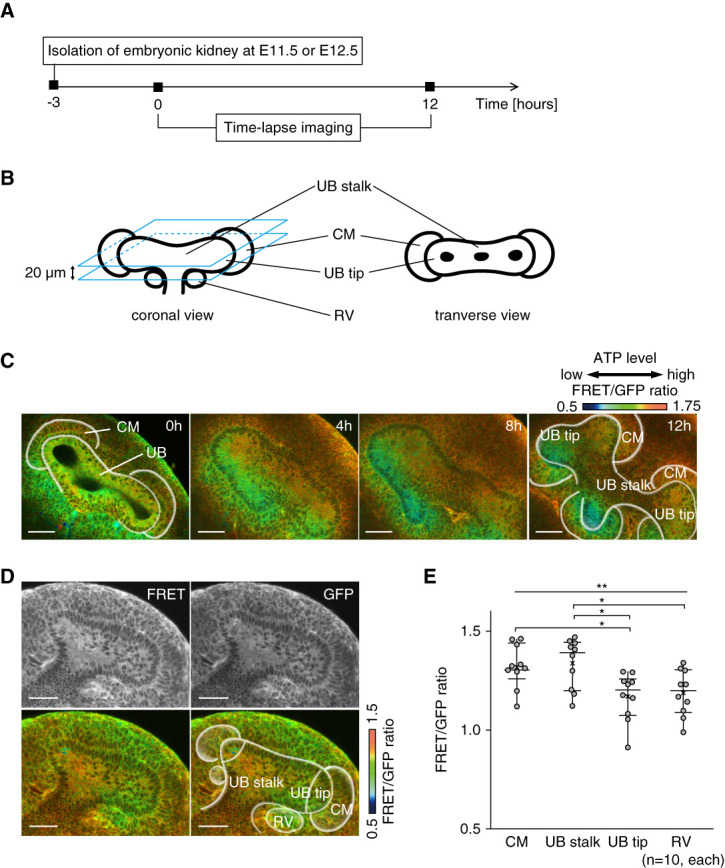
**Cytosolic ATP imaging in embryonic kidneys during branching morphogenesis.** (A) Experimental procedure: We isolated embryonic kidneys of GO-ATeam2 mice at E11.5 or E12.5, followed by *ex vivo* ATP imaging. (B) Schema of the UB, CM, and RV. A coronal or transverse view of dissected metanephric kidneys were observed depending on the orientation when placed on the culture membrane. The field of view could change over time during the culture process because the metanephric kidneys gradually enlarged. In each time-lapse session, *z*-stack images were collected at 5 *µ*m intervals (five optical slices, total depth 20 *µ*m) at the same observation area. (C) Representative images of real-time *ex vivo* imaging of embryonic kidney (E11.5) of GO-ATeam2 mice visualized the cytosolic ATP level and its changes in UB and CM cells. (0, 4, 8, 12 hours). (D) FRET, GFP, and ratio images (FRET/GFP) at E12.5 embryonic kidney. In the GO-ATeam2-expressing cells, the FRET/GFP fluorescence intensity ratio in each pixel correlates with the cytosolic ATP level, thereby allowing ATP monitoring. White lines show CM, UB stalk, UB tip, and RV. (E) Ratios (FRET/GFP) in CM, UB stalk, UB tip, and RV cells (*n*=10 per group). Cytosolic ATP levels varied by location. The arrangement of cells and their clustering made it possible to broadly distinguish CM, UB, and RV within the metanephric kidney. We analyzed the cytosolic ATP levels in CM, UB, and RV. Data are presented as median (25th–75th percentile). Statistical significance among CM, UB stalk, UB tip, and RV was assessed using one-way ANOVA with Bonferroni correction. *P < 0.05, **P < 0.01. Scale bars: 50 *µ*m (C and D). CM, cap mesenchyme; FRET, fluorescence energy transfer; GFP, green fluorescent protein; RV, renal vesicle; UB, ureteric bud.

MetaMorph software (Molecular Devices, San Jose, CA) was used for image analysis. In each area (CM, UB stalk, UB tip, and renal vesicle [RV]), the average intensity of the donor fluorophore (FRET) was divided by that of the acceptor fluorophore (GFP), to obtain the emission ratio. Ratio images were created as shown in the lower panels of Figure 1.

### Immunofluorescence Study

1. Cryo-sectioned kidney samples: Dissected kidneys from embryonic mice were briefly fixed in 4% paraformaldehyde and incubated in 20% sucrose. Optimal cutting temperature compound-embedded kidneys were cryosectioned into 6.0 um slices and mounted on glass slides (Matsunami Glass). The sections were blocked with blocking solution (5% normal goat serum, PBS) for 1 hour and incubated with primary antibodies overnight at 4°C. The following primary antibodies were used: anti-SIX Homeobox 2 (*Six2*; 1:500, 11562-1-AP; Proteintech), pan-cytokeratin (1:500, C2562; Sigma–Aldrich), cleaved Caspase 3 (Casp3) (1:200, 9664; Cell Signaling), and N-cadherin (1:100, ab18203; Abcam). After washing, sections were incubated with the corresponding secondary antibodies for 1 hour at room temperature.
2. Whole-mount kidney samples: Whole-mount immunostaining of cultured kidneys was performed as follows. Kidney samples were briefly fixed in 4% paraformaldehyde, incubated in a blocking solution for 1 hour, and then incubated with primary antibodies for 24–48 hours at 4°C. The antibodies used were anti-pan cytokeratin (1:500) and *Six2* (1:500) detailed above. The samples were rinsed in PBS containing 0.1% Triton X-100 for several hours and incubated with the corresponding secondary antibodies for several hours. Once the secondary antibodies were removed, the samples were washed several hours with PBS containing 0.1% Triton X-100.

All staining samples were analyzed using a confocal microscope (FV1000D, Olympus) or inverted two-photon microscope (FV1200MPE-IX83, Olympus). Whole-mount immunofluorescence imaging of embryonic kidneys was performed with the two-photon microscope using an excitation wavelength of 810 nm. Fluorescence signals were separated and detected with an infrared-cut filter (BA750RXD), two dichroic mirrors (DM505 and DM570), and two emission filters (BA495–540 nm for Alexa Fluor 488 and BA575–630 nm for Alexa Fluor 546, both from Olympus). *Z*-stack images were acquired and processed for three-dimensional reconstruction using Imaris software (Bitplane, Zurich, Switzerland).

### Transmission Electron Microscopy

Isolated metanephric kidneys at E12.5 were fixed with 2% glutaraldehyde followed by 1% osmium tetroxide postfixation and graded series of dehydration. Transitional acetone incubation was performed before gradual embedding into Epon. Ultrathin sections were analyzed using a transmission electron microscope (H7650 microscope, Hitachi).

### Quantification of mRNA by Real-Time Reverse Transcription PCR

RNA extraction and real-time reverse transcription PCR were performed as described previously.^36,37^ Primer sequences are listed in the Supplemental Table 1. The expression levels were normalized to *18S* rRNA expression.

### Statistical Analyses

We used the Mac statistical analysis (ver. 8.0; Esumi Corporation, Tokyo, Japan) for all statistical analyses. Data are shown as mean±SEM or median (25th–75th percentile). Statistical significance between the two groups was determined using an unpaired *t* test. One-way ANOVA with Bonferroni correction was used for multiple comparisons between three or four groups. The Jonckheere-Terpstra trend test was performed to demonstrate the dose-dependent response by 2-DG administration.

### Study Approval

All animal studies were approved by the Animal Research Committee, Graduate School of Medicine, Kyoto University, and were performed in accordance with the National Institutes of Health Guide for the Care and Use of Laboratory Animals.

## Results

### Spatiotemporal Dynamics of the Cytosolic ATP Levels During Kidney Development

As detailed in the Introduction, the nephron number mainly depends on the implementation of normal branching of the UB in the early phase (E11.5 to E15.5), which peaks at E13.5.^24^ Therefore, in this study, we analyzed embryonic kidneys obtained from GO-ATeam2 mice during E11.5–E12.5.

We successfully visualized cytosolic ATP levels at the single-cell level and investigated the spatiotemporal dynamics of cytosolic ATP levels in both UB and CM cells (Figure 1). Cytosolic ATP levels of metanephric kidney cells were analyzed 3 hours after the start of *ex vivo* culture because they are easily affected by mechanical stress and temperature during isolation (Figure 1A). In *ex vivo* imaging of the dissected metanephric kidney, we observed it in a coronal or transverse view depending on the orientation when placed on the culture membrane, and *z*-stack images were acquired at 5 μm intervals (five slices, total depth 20 μm) at the same observation area (Figure 1B). Cytosolic ATP dynamics of isolated metanephric kidneys (E11.5) were captured by real-time *ex vivo* imaging using multiphoton microscopy during branching morphogenesis under an explant culture system at 10-minute intervals over 12 hours (Figure 1, A–C, and Supplemental Movie 1). In the isolated metanephric kidney at E11.5, sequential images started from a transverse view at 0 hour (the far-left image in Figure 1C), capturing the initial bifurcation of the UB into two branches. During the 12-hour time-lapse imaging, each UB tip underwent further bifurcation, and the metanephric kidney gradually expanded in size. Using this system, we examined cytosolic ATP levels of each cell type. When all of the UB tip, UB stalk, CM, RV could not be simultaneously captured within a single focal plane, different *z*-stack layers were examined, and data were extracted from the appropriate optical sections (Supplemental Figure 1). The ATP levels of UB tip cells were significantly lower than those of UB stalk cells and CM cells capping UB tip (Figure 1, D and E). Furthermore, ATP levels in the RV were significantly lower than those in the UB stalk cells. The FRET/GFP ratio within the UB tip tended to be relatively higher in regions closer to the UB stalk, whereas UB regions adjacent to the RV showed lower ratios. Consistent with these spatial trends, the medullary side exhibited lower ratios than the cortical side, demonstrating regional heterogeneity in cytosolic ATP levels within the UB. To further assess whether cellular or nuclear architecture might influence the cytosolic ATP signal, we compared FRET/GFP ratios measured from (*1*) the entire cell cluster area and (*2*) multiple small regions of interest (ROIs; more than 20 per field) carefully defined to exclude nuclei within the same region. These two measurements yielded nearly identical values (Supplemental Table 2), suggesting that the potential contribution of nuclear density or size to the FRET/GFP ratio was negligible.

### Reduction of Cytosolic ATP Levels of Both UB Tip and CM Cells by Glycolytic Inhibition, But Not by OXPHOS Inhibition

**Figure 2:**
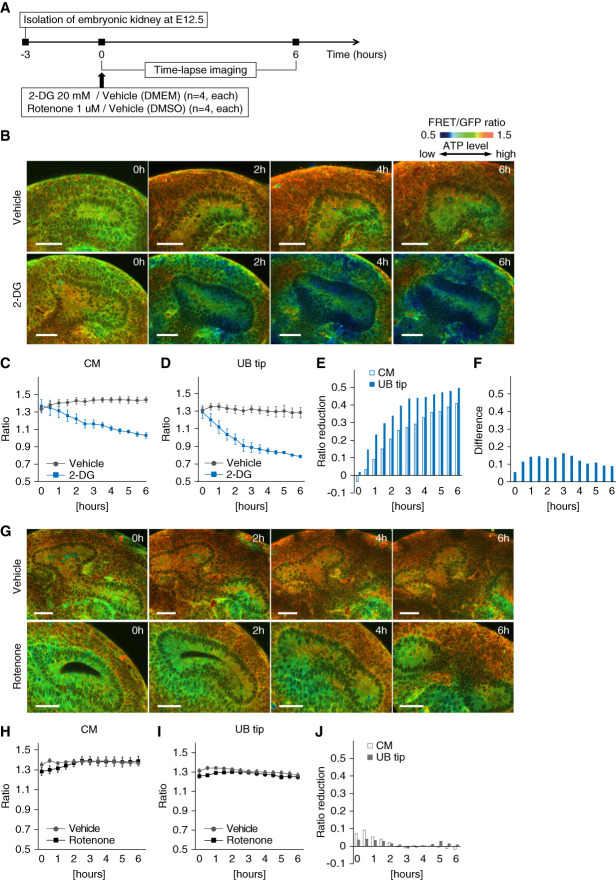
**Reduction of cytosolic ATP level by glycolytic inhibition.** (A) Experimental procedure: We isolated the GO-ATeam2 mice's embryonic kidney at E12.5 and then performed *ex vivo* ATP imaging after administration of 20 mM 2-DG (an inhibitor of glycolysis) and 1 *µ*M rotenone (an inhibitor of OXPHOS). (B) Real-time *ex vivo* imaging of GO-ATeam2 mice's embryonic kidney (E12.5) visualized the cytosolic ATP level and its changes in UB and CM cells after 2-DG administration (0, 2, 4, 6 hours). (C and D) Time course of ratios (FRET/GFP) in UB and CM cells after 2-DG administration. Data are shown as mean±SEM. (E) The graph shows a reduction in the ratio compared with the vehicle in UB and CM cells. The ratio reduction was calculated from the difference between the mean values of the vehicle and 2-DG treatment groups. (F) The difference in the ratios reduction between UB and CM cells was calculated on the basis of the average ratio reduction between UB and CM cells. The ratio reduction in UB cells was faster and more obvious than in CM cells. (G) Real-time *ex vivo* imaging of GO-ATeam2 mice's embryonic kidney (E12.5) visualized the cytosolic ATP level and its changes in UB and CM cells after rotenone administration (0, 2, 4, 6 hours). (H and I) Time course of the ratios (FRET/GFP) in UB and CM cells after rotenone administration. Data are shown as mean±SEM. (J) The graph shows a reduction in the ratio compared to the vehicle in UB and CM cells. The ratio reduction was calculated as the difference between the mean values of the vehicle-treated and rotenone-treated groups. 2-DG, 2-Deoxy-D-glucose; OXPHOS, oxidative phosphorylation.

Next, we investigated the temporal changes in the cytosolic ATP levels of UB tip and CM cells by inhibiting the ATP production pathways. We used inhibitors of glycolysis or OXPHOS to explore which metabolic pathway mainly contributes to ATP production during branching nephrogenesis, using *ex vivo* ATP imaging (Figure 2A). Administration of 2-DG to isolated embryonic kidneys (E12.5) of GO-ATeam2 mice significantly decreased the cytosolic ATP levels (FRET/GFP ratio) in both UB tip and CM cells (Figure 2, B–D), and the rate of decrease was more pronounced in UB tip cells (Figure 2E). Specifically, the FRET/GFP ratio in UB tip cells showed a rapid decline within the first 3 hours, decreasing by an average of 0.44 (approximately 34%), followed by a further slight reduction of an additional 0.06 (average total reduction of 0.50, approximately 40%) by 6 hours (Figure 2E). By contrast, CM cells exhibited a smaller and more gradual decrease, with a total average reduction of 0.41 (approximately 30%) at 6 hours. Furthermore, the difference between UB tip and CM cells in the rate of reduction in ATP levels during 2-DG treatment increased within the first hour (Figure 2F). These findings indicate that ATP levels in UB tip cells decreased more rapidly than those in CM cells. Time-lapse imaging of embryonic kidneys also suggested the cessation of UB elongation after administration of 2-DG (Figure 2B and Supplemental Movie 2), which might lead to the attenuation of UB branching.

Conversely, the administration of rotenone, an inhibitor of OXPHOS did not change ATP levels in either UB tip nor CM cells (Figure 2, G–J). These results indicate that glycolysis substantially contributes to ATP production in both UB and CM cells, especially in UB tip cells during the early phase of development.

### Loss of N-cadherin Expression and Lateral Integrity of CM Cells by Glycolytic Inhibition, But Not by OXPHOS Inhibition

**Figure 3:**
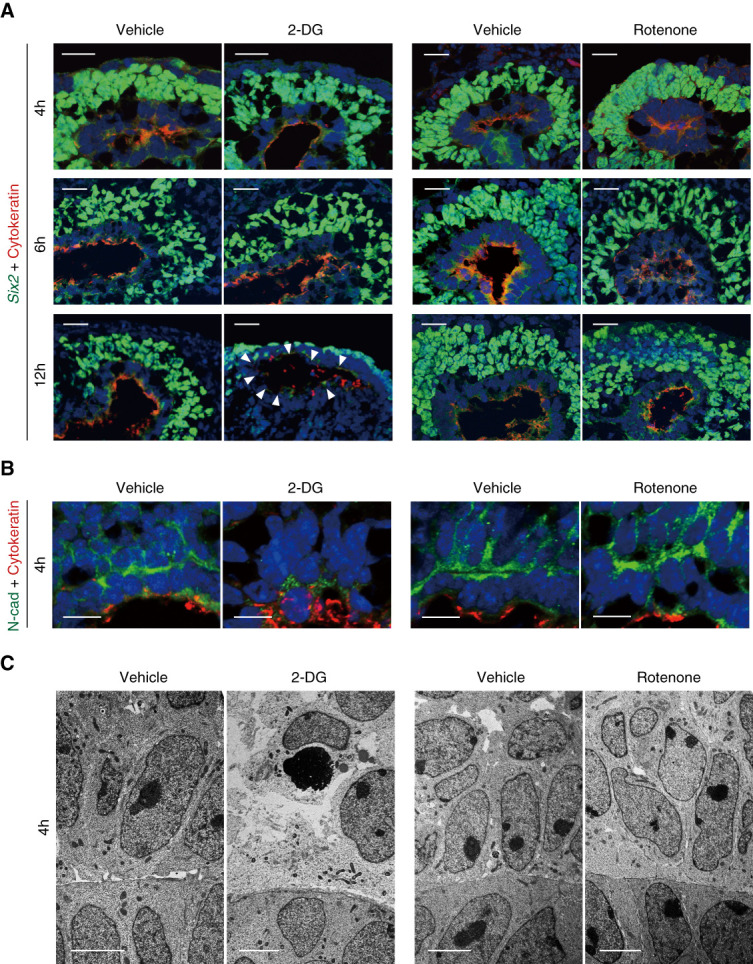
**Alterations of morphology and specific markers' expression of UB and CM cells after 2-DG and rotenone treatment.** (A) Time course (4, 6, and 12 hours after 2-DG or rotenone treatment) of specific marker expression in UB (pan-cytokeratin, red) and CM (*Six2*, green) cells after 2-DG and rotenone treatment. Arrowheads indicate decreased expression of pan-cytokeratin signal in UB cells. (B) Expression of N-cadherin (N-cad, green) and pan-cytokeratin (red) 4 hours after 2-DG and rotenone treatment. (C) Electron microscopy images of embryonic kidneys 4 hours after 2-DG and rotenone treatment. Scale bars: A, 20 *µ*m; B, 10 *µ*m; C, 5 *µ*m. *Six2*, SIX Homeobox 2.

As mentioned previously, UB branching depends on the interaction between UB and CM. To explore phenotypic changes in UB and CM cells by glycolytic inhibition, we analyzed the expression of specific markers for each cell type in cultured E12.5 embryos under glycolytic inhibition or OXPHOS inhibition. Pan-cytokeratin was used as a marker for UB and *Six2* as a marker for CM. In the 2-DG treated group, both pan-cytokeratin and *Six2* expression were maintained after 6 hours of treatment (Figure 3A), but after 12 hours, *Six2*-positive mesenchyme cells were clearly reduced, and pan-cytokeratin signal in UB cells was diminished (Figure 3A, arrowheads). Interestingly, 4 hours after treatment, the expression of neural cadherin (N-cadherin), a cell adhesion molecule that plays an important role in lateral intercellular adhesion in the metanephric mesenchyme,^38,39^ was significantly decreased (Figure 3B). Electron microscopy images showed loosening and detachment of cell-cell adhesion and irregular cell alignment in CM cells 4 hours after 2-DG administration (Figure 3C), suggesting loss of lateral integrity of CM cells. By contrast, the rotenone-treated group showed no significant differences in the expression of specific markers compared with the vehicle group (Figure 3, A and B). Electron microscopy findings also showed that cell-cell adhesion was maintained in the rotenone-treated group (Figure 3C).

**Figure 4:**
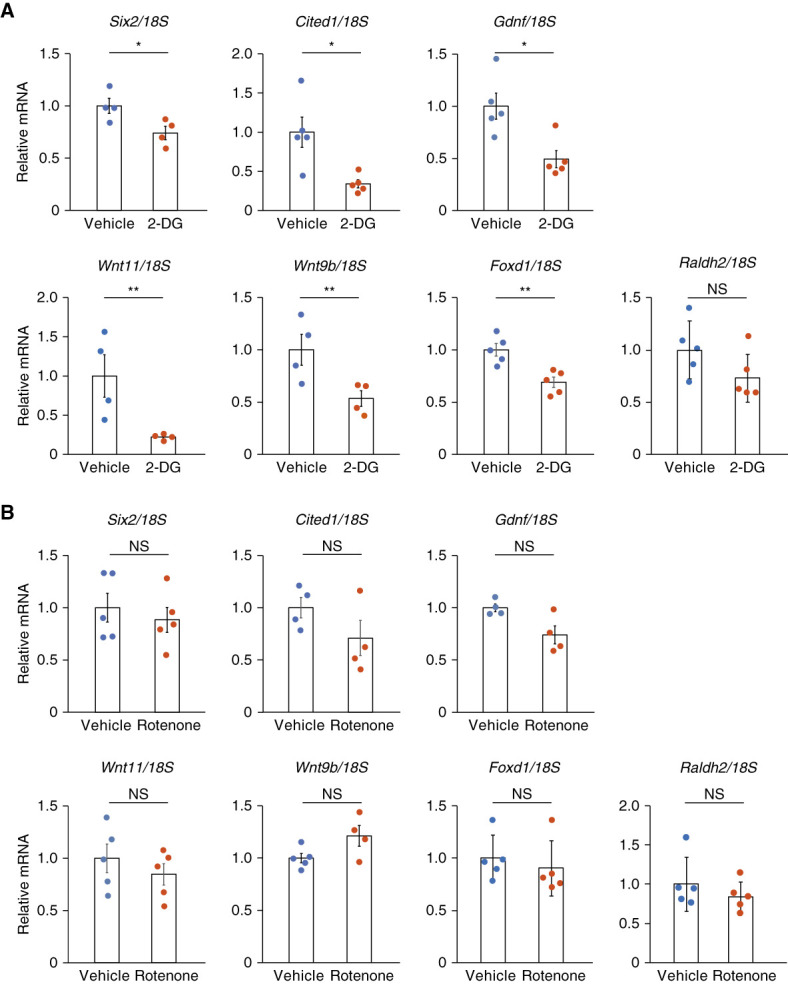
**Relative mRNA expression level of specific markers for CM, UB cells, and stromal cell markers after 2-DG and rotenone treatment.** Relative mRNA expression of *Six2*, *Cited1*, *Gdnf*, *Wnt11*, *Wnt9b*, *Foxd1*, and *Raldh2* after 6 hours (A) 2-DG or (B) rotenone treatment compared with vehicle (*n*=4 or 5, each group). Data are shown as mean±SEM. Statistical significance was determined by unpaired *t* test. *P < 0.05, **P < 0.01. *Cited1, Cbp/p300-interacting transactivator 1; Foxd1, Forkhead Box D1; Gdnf, glial cell-derived neurotrophic factor; Raldh2, retinaldehyde dehydrogenase 2*.

Next, we examined the mRNA expression of UB and CM cell markers in embryonic kidneys. While protein expression of CM and UB markers (*Six2* and pan-cytokeratin) was still unchanged at 6 hours post 2-DG administration (Figure 3A), mRNA expression of *Six2*, *Cbp/p300-interacting transactivator 1* (*Cited1*, a marker for immature mesenchyme cells), and *glial cell-derived neurotrophic factor* (*Gdnf*, a major UB attractant in CM cells) at 6 hours post 2-DG administration was significantly reduced (Figure 4A), leading to a reduction of CM markers after 12 hours (Figure 3A). In addition, the expression of *Wnt11* (a marker of the tip of branching UB^40^) and *Wnt9b* (a marker of UB, which is more pronounced in the stalk region than in the tips^41^) was also markedly reduced (Figure 4A). By contrast, the rotenone-treated group showed no significant differences in the expression of these markers compared with the vehicle group (Figure 4B). We also checked the mRNA expression of the stromal cell marker (*Forkhead Box D1* [*Foxd1*]) and retinoic acid signaling marker (*retinaldehyde dehydrogenase 2* [*Raldh2*]) because previous studies indicated that retinoid-dependent stromal cell signals act on UB/CM interaction.^42,43^ While *Raldh2* expression did not change between groups, *Foxd1* expression was significantly reduced in the 2-DG group compared with the vehicle-treated groups (Figure 4A). The expression of these markers did not change in the rotenone-treated group, either (Figure 4B).

### Dose-Dependent Suppression of UB Branching by Glycolytic Inhibition, But Not by OXPHOS Inhibition

**Figure 5:**
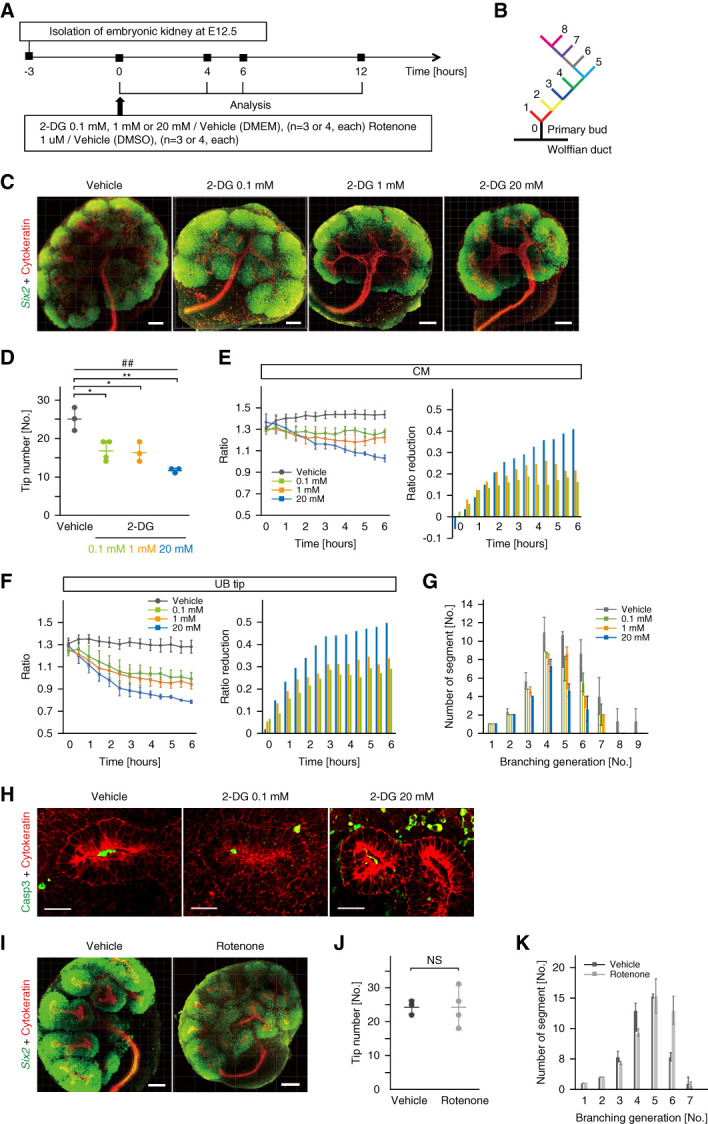
**Suppression of UB branching by glycolytic inhibitor.** (A) Experimental procedure: We isolated metanephric kidneys (E12.5) of wild-type or GO-ATeam2 mouse embryos, and then, 12 hours later, examined UB branching after administration of 2-DG or rotenone. (B) Colored lines used to indicate the generation of branches according to the following conventions: The primary bud from the Wolffian duct is considered as zero (black); the first two branches that form from the primary bud are the first branching generation (red); the second generation is yellow; third generation is dark blue; fourth generation is green; fifth generation is light blue; sixth generation is gray; seventh generation is purple; and eighth generation is pink. (C and D) Representative two-photon microscopy images of the embryonic kidneys (C, *Six2*; green, pan-cytokeratin; red) and tip number (D) in each group; we compared the UB branching after low-dose (0.1 or 1 mM) and high-dose (20 mM) 2-DG administration. Statistical significance among vehicle and 2-DG treatment groups was assessed by one-way ANOVA with Bonferroni correction (*P < 0.05, **P < 0.01) and the Jonckheere-Terpstra trend test (^##^*P* < 0.01). (E and F) Time courses of ratio reduction in UB and CM cells after 2-DG treatment are shown in the graphs. Ratio reduction was calculated from the difference between the mean value of the vehicle group and the 2-DG (0.1, 1 and 20 mM) treatment groups. (G) The number of branch segments shown in Figure 5B plotted on the horizontal axis. The vertical axis of left shows the total number of branch segments per order. The total number of branch segments increased exponentially and decreased toward the right end after reaching each peak. The right tail represents the maximum number of branching generations for each group. The AUC represents the total number of segments per kidney. (H) Representative images of cleaved Casp3 staining (green) with pan-cytokeratin (Cytokeratin, red) after 2-DG treatment. Casp3–positive cells markedly increased in the 20 mM group, but not in the 0.1 mM group compared with the Vehicle group. (I and J) Representative images of the embryonic kidneys (I, *Six2*; green, pan-cytokeratin; red) and tip number (J) in each group after rotenone treatment. (K) The number of branch segments plotted on the horizontal axis after rotenone treatment. The left vertical axis plots the total number of branch segments per order. Scale bars: 100 *µ*m (C and I), 20 *µ*m (H). AUC, area under the curve; Casp3, Caspase-3.

We next investigated whether glycolytic inhibition affects UB branching and whether the effect is dose-dependent. We analyzed the isolated embryonic kidneys (E12.5) of wild-type embryos treated with 2-DG (0.1, 1 and 20 mM) for 12 hours (Figure 5A). We calculated the number of branching generations and each branch segment, in addition to UB tips, as previously described.^24,44^ The UB outgrowth from the Wolffian duct is the primary bud defined as numbered zero (*0*). The first two branching from primary bud is the first generation (*1*) of branching (Figure 5B). The number of UB tips was significantly lower in the 2-DG–treated groups than in the vehicle-treated group (Figure 5, C and D). Moreover, the suppression of UB branching exhibited a dose-dependent response to glycolytic inhibition (*P* < 0.01, Jonckheere-Terpstra trend test, Figure 5D).

We also analyzed cytosolic ATP levels in the embryonic kidneys of GO-ATeam2 mice after the administration of various concentrations of 2-DG. The reduction in cytosolic ATP levels by the glycolytic inhibitor suggested a dose-dependent effect (Figure 5, E and F). ATP reduction in both UB tip and CM cells was more pronounced in the high-dose (20 mM) 2-DG–treated group. In CM cells, the FRET/GFP ratio decreased modestly by an average of 0.10–0.12 (8%–9%) within the first hour in both the 0.1 and 1 mM 2-DG groups, reaching a plateau thereafter at 1 hour in the 0.1 mM group. By contrast, in the 1 mM group, the ratio continued to decline gradually over the subsequent several hours, reaching an apparent plateau around 4 hours. The total reduction at 6 hours was approximately 0.16 (12%) and 0.22 (17%) in these two groups, respectively. In the 20 mM group, ATP levels continued to decline gradually beyond 3 hours, reaching a total reduction of about 0.41 (30%) at 6 hours. By comparison, UB tip cells exhibited a much steeper and dose-dependent decrease during the initial 3 hours, with average reductions during the first hour of 0.16 and 0.19 (12%–15%) in the 0.1 and 1 mM groups and 0.23 (18%) in the 20 mM group. This rapid decline persisted for 3 hours, after which ATP levels became nearly stable or showed only a slight further decrease, resulting in total reductions at 6 hours of approximately 0.29 (22%), 0.34 (26%), and 0.50 (40%) in the 0.1, 1, and 20 mM groups, respectively (Figure 5, E and F).

Consequently, the maximum branching order (the right tail in each group in Figure 5G) was reduced from nine in the vehicle group to six in the high-dose group, and the total number of branch segments per kidney (area under the curve [AUC]) was significantly reduced in the high-dose group compared with the vehicle group (Vehicle: 46.0±3.5, 20 mM: 21.7±0.7, *P* < 0.01, unpaired *t* test; Figure 5G). Furthermore, the reduction in the total number of branch segments per kidney (AUC) showed a dose dependent effect (Vehicle: 46.0±3.5, 0.1 mM: 31.0±2.9, 1 mM: 30.3±2.3, 20 mM: 21.7±0.7, *P* for trend <0.01, Jonckheere-Terpstra trend test).

To further determine whether the observed branching suppression by glycolytic inhibition was associated with cell death, we performed immunostaining for cleaved Casp3. A marked increase in Casp3–positive cells was detected in the 20 mM 2-DG–treated group, whereas no apparent increase was observed in the 0.1 mM groups (Figure 5H). These results indicate that high-dose 2-DG induced apoptosis possibly because of severe ATP depletion, whereas low-dose 2-DG suppressed UB branching without overt cytotoxicity.

By contrast, there were no significant differences in ATP levels or UB branching between the rotenone-treated group and the vehicle group (DMSO group; Figure 5, I–K). These findings indicate that glycolytic inhibition reduced cytosolic ATP levels in both UB and CM cells, suppressed UB branching, and led to a low nephron number.

## Discussion

Maternal nutritional status has been implicated in determining nephron number, highlighting the critical role of energy metabolism in kidney development. During kidney development, diverse cell populations undergo proliferation, differentiation, and complex morphogenesis, while their underlying energy metabolism remains largely unexplored. In this study, we successfully performed time-lapse *ex vivo* imaging of embryonic kidneys from GO-ATeam2 mice using multiphoton microscopy, enabling a detailed analysis of single-cell ATP dynamics during branching nephrogenesis. Previous studies have assessed ATP levels and metabolic profiles in cultured cells and in organs using luciferase assays and the Extracellular Flux Analyzer system. However, these approaches are unable to examine spatiotemporal ATP dynamics across distinct cell populations. Here, we simultaneously visualized intracellular ATP levels among different cell types, including UB and CM cells, enabling a direct comparison of ATP levels in these cells.

This methodology first revealed that ATP levels in UB tip cells were significantly lower than those in UB stalk cells and CM cells, and that ATP levels in RV cells were also significantly lower than those in UB stalk cells. As mentioned above, developing cells undergo rapid proliferation and differentiation, requiring substantial energy for lipid, protein, and nucleotide synthesis.^45^ Accordingly, the low ATP levels observed in UB tip cells and RV cells may suggest larger ATP consumption exceeding ATP synthesis.

Next, we confirmed that glycolysis inhibition led to a significant reduction in ATP levels in both UB tip and CM cells. By contrast, OXPHOS inhibition through Complex 1 did not show a significant reduction in ATP levels, suggesting that glycolysis is the predominant energy source during the early stages of metanephric development. In general, undifferentiated and proliferating cells during development enhance glucose uptake, activate glycolysis and related pathways, including the pentose phosphate and hexosamine pathways, and facilitate ATP and nucleotide production.^46–48^ Consistent with this notion, previous studies have reported strong hypoxia-inducible factor (HIF)-1*α* expression in UB tip cells under physiologically hypoxic conditions.^49^ HIF-1*α* acts as a master regulator of glycolytic enzymes and promotes branching morphogenesis by facilitating metabolic adaptation to hypoxia. Therefore, the dose-dependent suppression of UB branching observed after 2-DG treatment in this study further supports the importance of HIF-dependent glycolytic regulation in UB development. Although glycolysis is less efficient in ATP production compared to OXPHOS, it has the advantage of generating energy at a faster rate while limiting excessive reactive oxygen species accumulation.^50^ In addition, undifferentiated cells exhibit immature mitochondrial structures, low membrane potential, and underdeveloped electron transport chain functions.^47,51^ Consequently, undifferentiated and proliferating cells rely on aerobic glycolysis, known as the Warburg effect, as their primary metabolic strategy.^52^ Previous studies have suggested that NPCs at the early stage of metanephric development (E13.5) exhibit a greater dependence on glycolysis than late-stage NPCs (E19.5 or P0).^25^ Our results demonstrated that, in addition to CM cells, UB cells also exhibited a strong dependence on glycolysis. Furthermore, ATP depletion induced by glycolysis inhibition occurred more rapidly and markedly in UB tip cells than in CM cells. While previous study showed the importance of pyruvate in UB branching,^53^ our study is the first to show that UB cells rely on glycolysis and that UB tip cells exhibit greater dependence on glycolysis than CM cells.

Branching morphogenesis progresses through interactions between UB cells and CM cells. In this study, we found that glycolysis inhibition resulted in a significant reduction in the expression of UB-specific and CM-specific markers. Along with the decreased expression of *Six2* and *Cited1*, which are crucial for maintaining the NPC niche and are expressed in CM cells, the expression of *Gdnf*, *Wnt11*, and *Wnt9b*, which play key roles in UB-CM interactions, was also markedly reduced.^40,54,55^ By contrast, OXPHOS inhibition had no effect on the expression of these UB-specific and CM-specific markers, consistent with unchanged ATP levels.

ATP levels in both CM and UB were markedly reduced within 3 hours after 2DG administration, and following this decrease in ATP, the gene expression of CM-specific and UB-specific markers was significantly reduced at 6 hours post-treatment. Moreover, although immunostaining for *Six2* and Pan-cytokeratin showed no obvious reduction at 6 hours, their protein expression was markedly diminished at 12 hours. These findings suggest that the intracellular ATP reduction caused by glycolysis inhibition serves as a critical trigger for these sequential alterations.

Furthermore, 4 hours after 2-DG administration, reduced N-cadherin expression, and disorganized alignment of CM cells were evident by electron microscopy, suggesting a loss of their lateral integrity. These observations indicate that CM cells are also dependent on glycolysis. The underlying mechanism may involve both a direct effect of glycolysis inhibition on CM cells and an indirect effect mediated through altered UB cells. Although no previous studies have reported that glycolysis inhibition directly reduces ATP levels in NPCs, *in vitro* experiments using mesenchymal stem cells have shown that glycolysis inhibition leads to ATP depletion in mesenchymal stem cells.^56^ These findings support the idea that the ATP reduction observed in CM cells after 2-DG treatment may represent a direct effect of glycolysis inhibition. Conversely, the ATP reduction observed in CM cells may also be a secondary effect mediated by UB cells. *Wnt11*, which is highly expressed specifically in UB tip cells,^40^ has been suggested to play a crucial role in maintaining and regulating the function of the NPC niche. In *Wnt11* mutant mice, the polarity of CM cells surrounding the UB is disrupted, ultimately leading to a reduced number of nephrons.^57^ In this study, we found that ATP levels in UB tip cells were markedly reduced shortly after 2-DG administration, accompanied by a significant decrease in *Wnt11* mRNA expression. Because intracellular ATP depletion is generally known to suppress gene transcription by RNA polymerase and reduce mRNA levels,^58,59^ rapid loss of ATP in UB tip cells might also contribute to the downregulation of *Wnt11* expression, which in turn may have secondarily compromised the lateral integrity of CM cells. High-dose 2-DG treatment markedly increased Casp3–positive cells in the CM and stromal regions, whereas the UB epithelium showed no obvious increase. By contrast, even mild inhibition of glycolysis suppressed UB branching without evident cytotoxicity, suggesting that limited glycolytic ATP supply is sufficient to compromise energy-dependent morphogenetic processes. Taken together, our results show that glycolysis inhibition reduces UB branching and tip formation in a dose-dependent manner, strongly supporting that glycolysis is crucial for fueling UB branching.

This study has the following limitation. Owing to the technical challenges associated with *in vivo* imaging of fetal kidneys, our analyses were performed using *ex vivo* metanephros cultures. Therefore, it remains unclear whether the ATP dynamics observed in this study fully recapitulate those occurring *in vivo* because the system lacks *in vivo* factors such as blood flow, systemic energy status, and maternal-fetal nutrient exchange and provides a relatively hyperoxic environment compared with the physiological intrauterine condition, all of which may influence metabolic activity and cellular behavior. Future studies using more physiological, low-oxygen (hypoxic) culture conditions will be necessary to better reproduce the metabolic environment of the developing kidney. Nevertheless, as *ex vivo* analysis is widely used in studying fetal kidney development, the observed ATP dynamics are considered to reflect physiologically relevant processes with reasonable reliability.

In conclusion, we successfully visualized single-cell ATP dynamics during kidney development using *ex vivo* live imaging of GO-ATeam2 mice. Our findings demonstrate that glycolysis-dependent energy supply plays a crucial role in branching morphogenesis during kidney development. This study provides important insights into the metabolic regulation of nephrogenesis and offers a significant implication in understanding the relationship between the intrauterine environment and kidney development.

## Data Availability

All original data, including deidentified patient-level data or individual laboratory data measurements, are included in the manuscript and/or supplemental material.

## Declarative Statements

All animal experiments were conducted in accordance with the NIH Guide for the Care and Use of Laboratory Animals or an equivalent standard that meets or exceeds the ethical and welfare requirements outlined in the NIH Guide. All protocols were approved by the appropriate institutional animal care and use committee.

## Supplemental Material

Supplemental Figure 1. Representative *z*-stack images illustrating the optical plane assignment and workflow for region-specific ATP quantification in the metanephric kidney. (A) In the original imaging setup, *z*-stack images were acquired at 5 *µ*m intervals (five slices in total, covering a depth of 20 *µ*m) at the same observation point. Schematic representation of the coronal view showing the relative positions of optical slices. Slice 1 corresponds to the superficial plane near the tissue surface, containing the UB tip and CM, whereas Slice 4, located 15 *µ*m below Slice 1, includes the UB stalk and RV. (B) Representative FRET, GFP, and ratio images at Slice 1 and Slice 4. Red dotted lines indicate ROIs used for cytosolic ATP quantification. For each region, ATP levels were measured using two complementary approaches: (*1*) a large ROI encompassing the entire target region and (*2*) multiple small ROIs (≥20 per region) manually placed to exclude nuclei. Comparable FRET/GFP ratios obtained by both methods confirmed that nuclear content did not significantly influence cytosolic ATP quantification in this imaging system (see Supplemental Table 2). Scale bars: 50 *µ*m.

Supplemental Table 1. Primer sequences used for real-time PCR.

Supplemental Table 2. Comparison of the FRET/GFP ratio measurements with and without nuclear exclusion.

Supplemental Movie 1. 12-hour time-lapse ATP imaging of the UB branching (E11.5).

Supplemental Movie 2. 6-hour time-lapse ATP imaging of embryonic kidney after 2-DG treatment (E12.5).
